# Bifunctional metal nanoparticles in agriculture: an opinion on their role as fungicides and fertilizers

**DOI:** 10.3389/fpls.2026.1821016

**Published:** 2026-06-03

**Authors:** Jesusita Rosas-Diaz, Vianii Cruz-López, Cecilia Hernández-Ramírez, Eric G Echeverría-Pérez, María J. Martínez-Carreón, Pastor T. Matadamas-Ortiz, Heriberto Cruz-Martínez

**Affiliations:** 1 Tecnológico Nacional de México, Instituto Tecnológico del Valle de Etla, Santiago Suchilquitongo, Oaxaca, Mexico; 2 Facultad de Ciencias Físico-Matemáticas, Universidad Autónoma de Nuevo León, San Nicolas de los Garza, Nuevo León, Mexico; 3 Instituto Politécnico Nacional, Centro Interdisciplinario de Investigación para el Desarrollo Integral Regional Unidad Oaxaca, Santa Cruz Xoxocotlán, Oaxaca, Mexico

**Keywords:** crop protection, metal nanoparticles, metal oxide nanoparticles, micronutrient sources, nanomaterials

## 1 Introduction

Agriculture faces significant challenges, including diseases that reduce crop yields and deficiencies in essential soil nutrients that limit plant growth, decrease productivity, and compromise global food security (Paramesh et al., 2023; Gai and Wang, 2024). Currently, these challenges are addressed through the intensive use of conventional synthetic agrochemicals. However, their excessive or improper application can lead to soil degradation, water contamination, biodiversity loss, pest resistance, and potential risks to human health and environmental sustainability (Aktar et al., 2009; Rani et al., 2021). Consequently, various environmentally friendly options have been studied (Backer et al., 2018; Fasusi et al., 2021; Cucu et al., 2025; Adkar-Purushothama et al., 2026), with particular emphasis on the application of nanomaterials as nanofungicides to control crop diseases caused by phytopathogenic fungi and as nanofertilizers that serve as micronutrient sources to enhance the growth and productivity of economically important crops (Fu et al., 2020; Ogwu and Izah, 2025). The growing interest in nanomaterials is primarily due to their unique physicochemical properties, which differ significantly from those of their bulk counterparts. Owing to their nanoscale size, high surface area, and enhanced reactivity, nanomaterials can interact efficiently with biological systems. These characteristics enable their effective application as nanofungicides and nanofertilizers in agriculture (Fu et al., 2020; Ogwu and Izah, 2025).

In recent years, nanomaterials —including nanocomposites, nanocarbons, nanogels, nanocapsules, and nanoemulsions—have emerged as promising alternatives for controlling phytopathogenic fungi and as advanced fertilizers. Among these, metal nanoparticles have attracted considerable attention due to their outstanding properties for these applications (Fu et al., 2020; Ogwu and Izah, 2025). To date, numerous studies have investigated the use of metal nanoparticles as fungicides or as fertilizers, and these findings have been extensively discussed across a wide range of review articles (Cruz-Luna et al., 2021; Fatima et al., 2021; Ndaba et al., 2022; Cruz-Luna et al., 2023; Kumar Balu et al., 2023; Sanguiñedo et al., 2023; Raza et al., 2024; Echeverría-Pérez et al., 2025; Shashidhara et al., 2025; Hernández-Ramírez et al., 2026). However, these review articles have predominantly analyzed each application separately. Recently, the potential of bifunctional metal nanoparticles in agriculture has gained increasing attention, as these materials can simultaneously function as nanofertilizers and nanofungicides. This bifunctionality offers a more efficient and potentially sustainable strategy, particularly for economically important crops, by integrating plant protection and nutritional supplementation into a single nanoparticle-based treatment. To date, the potential of bifunctional metal nanoparticles has been investigated in different crops (Zakharova et al., 2019; Keerthana et al., 2021; Lopez-Lima et al., 2021; Tryfon et al., 2021; Tamil Elakkiya et al., 2022; Deng et al., 2023; El-Sayed et al., 2023; Shams et al., 2023; Singh et al., 2023; Wee et al., 2023; Ajiwe and Popoola, 2024; Deng et al., 2024; Elbasuney et al., 2024; Ashraf et al., 2025; Lopez-Lima et al., 2025; Mochi et al., 2025; Saikia et al., 2026). Among them, tomato (*Solanum lycopersicum*) is the most extensively studied crop (Lopez-Lima et al., 2021; Deng et al., 2023; Shams et al., 2023; Ajiwe and Popoola, 2024; Deng et al., 2024; Ashraf et al., 2025), likely due to its high economic importance and significant nutritional value (Echeverría-Pérez et al., 2025). However, to the best of our knowledge, no review or opinion article has specifically and comprehensively addressed the simultaneous use of metal nanoparticles as both fungicides and fertilizers. Therefore, this opinion article provides a critical assessment of current research and discusses future perspectives on the development and application of bifunctional metal nanoparticles that can act simultaneously as fungicides and fertilizers in agricultural systems, using tomato as a model crop because it has been extensively evaluated in this context.

## 2 Advances in bifunctional metal nanoparticles as fungicides and fertilizers

Bifunctional metal nanoparticles represent an innovative strategy to simultaneously act as fungicides and fertilizers in agricultural systems. Metal nanoparticles can exhibit antifungal activity against phytopathogenic fungi, thus reducing disease incidence. At the same time, these metals are essential micronutrients that, in controlled concentrations, support key physiological processes such as photosynthesis and enzyme synthesis. This bifunctional approach could reduce reliance on conventional agrochemicals, optimize nutrient use efficiency, and promote more sustainable agriculture. To date, the potential of bifunctional metal nanoparticles has been investigated in different crops such as bean (*Phaseolus vulgaris*) (El-Sayed et al., 2023), eggplant (*Solanum melongena*) (Elbasuney et al., 2024), okra (*Abelmoschus esculentus*) (Keerthana et al., 2021), coffee (*Coffea arabica*) (Lopez-Lima et al., 2025), mung bean (*Vigna radiata*) (Mochi et al., 2025), tea (*Camellia sinensis*) (Saikia et al., 2026), mustard (*Brassica nigra*) (Tamil Elakkiya et al., 2022), lettuce (*Lactuca sativa*) (Tryfon et al., 2021), wheat (*Triticum aestivum*) (Zakharova et al., 2019), watermelon (*Citrullus lanatus*) (Deng et al., 2023), maize (*Zea mays*) (Singh et al., 2023; Wee et al., 2023), and tomato (*Solanum lycopersicum*) (Lopez-Lima et al., 2021; Deng et al., 2023; Shams et al., 2023; Ajiwe and Popoola, 2024; Deng et al., 2024; Ashraf et al., 2025). Among these, tomato is the most extensively studied crop. Therefore, we present advances in the bifunctional use of metal and metal oxide nanoparticles, demonstrating their effectiveness in agricultural applications using tomato as a model crop.

### 2.1 Metal nanoparticles

#### 2.1.1 Cu nanoparticles

**Figure 1:**
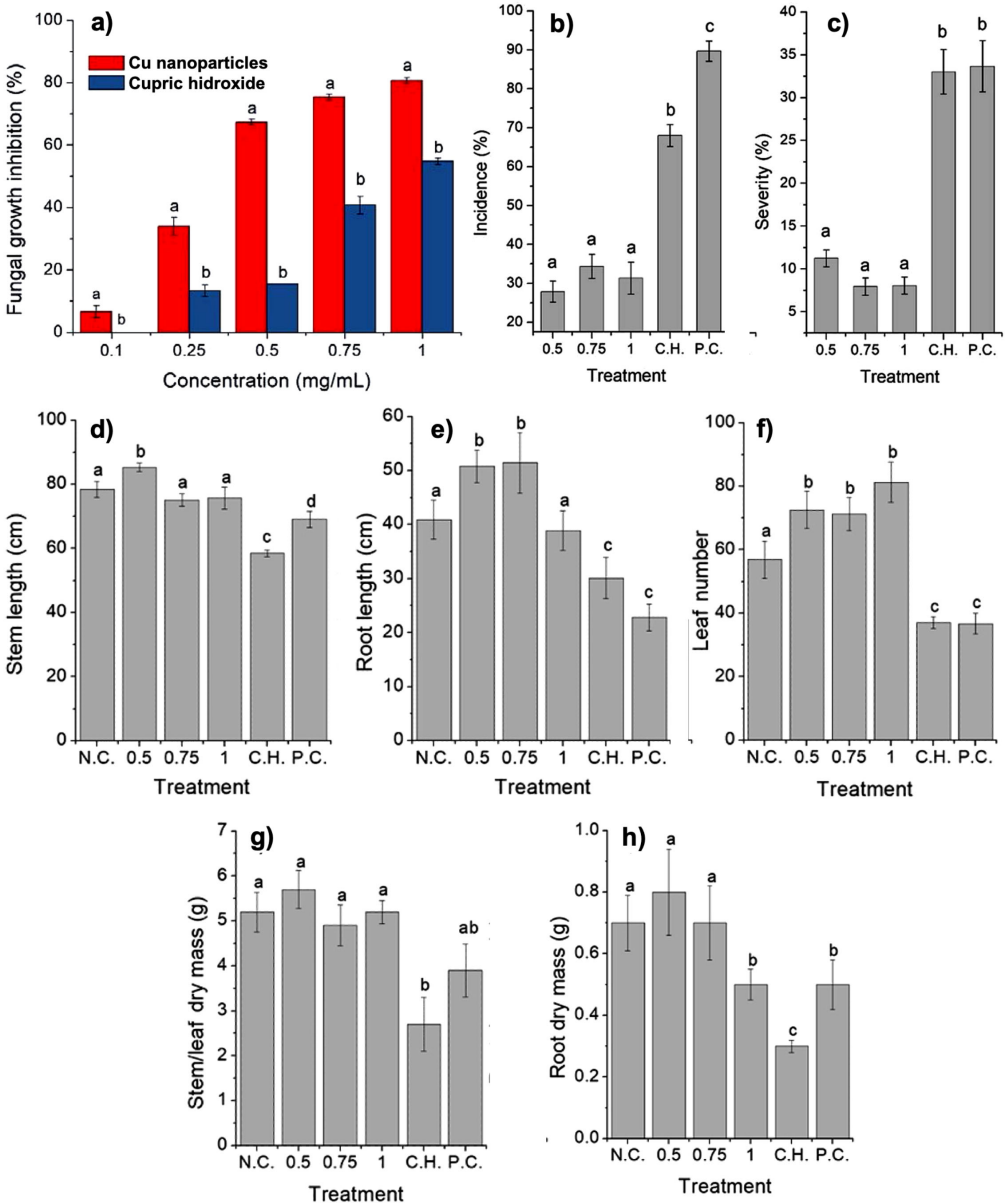
**(a)** Fungal growth inhibition under *in vitro* conditions of *Fusarium oxysporum* f sp. *lycopersici* exposed to Cu nanoparticles and cupric hydroxide at concentrations of 0.1, 0.25, 0.5, 0.75, and 1 mg/mL. Evaluation of the antifungal efficacy under *in vivo* conditions: **(b)** incidence and **(c)** severity of Cu nanoparticles for *Fusarium* wilt on plants 60 days after inoculation at concentrations of 0.5, 0.75, and 1 mg/mL. Effect of different treatments on various growth parameters: **(d)** stem length, **(e)** root length, **(f)** leaf number, **(g)** stem/leaf dry mass, and **(h)** root dry mass of plants 60 days post-inoculation with *Fusarium oxysporum* treated with Cu nanoparticles at concentrations of 0.5, 0.75, and 1 mg/mL. N.C., Negative control, C.H., Cupric hydroxide (Cupravit^®^ Hidro at 1 mg/mL), and P.C., Positive control. Bars with the same letters indicate values that are not significantly different according to Tukey’s test (p <0.05). Modified from Lopez-Lima et al., 2021.

Some studies have investigated the bifunctionality of Cu nanoparticles as fungicides and nanofertilizers (Lopez-Lima et al., 2021; Ajiwe and Popoola, 2024). For instance, Cu nanoparticles were used to control *Fusarium oxysporum* f. sp. *lycopersici* and promote tomato plant growth (Lopez-Lima et al., 2021). The Cu nanoparticles were produced via a green synthesis method, with sizes ranging from 200 to 500 nm. **Figure 1a** shows that the antifungal activity of the Cu nanoparticles under *in vitro* conditions was higher than that of cupric hydroxide at all studied concentrations (0.1, 0.25, 0.5, 0.75, and 1 mg/mL). For Cu nanoparticles at 1 mg/mL, antifungal activity exceeded 80% (**Figure 1a**). Subsequently, the antifungal efficacies under *in vivo* conditions (tomato plants of 60 days post-inoculation with *Fusarium oxysporum*) of the Cu nanoparticles (concentrations of 0.5, 0.75, and 1 mg/mL) were compared against copper hydroxide (commercial product at 1 mg/mL) and the positive control (infected with *Fusarium oxysporum* but without any antifungal). For tomato plants treated with Cu nanoparticles, the incidence (**Figure 1b**) and severity (**Figure 1c**) of the disease were significantly reduced compared with copper hydroxide and the positive control. Simultaneously, the effects of Cu nanoparticles on various plant growth parameters were investigated. For Cu nanoparticle treatments, most growth parameters were higher compared to the negative control (tomato plants unaffected by *Fusarium oxysporum* and untreated with any antifungal). The best results were obtained with 0.5 mg/mL Cu nanoparticles, where most growth parameters (stem length, stem/leaf dry mass, and root dry mass) increased compared with the negative control (**Figures 1d–h**). This improvement can be attributed to an optimal Cu supply that enhances photosynthesis, enzymatic activity, and biomass accumulation without inducing phytotoxicity. At higher concentrations, excess Cu may disrupt metabolic processes, limiting further growth improvement (Lopez-Lima et al., 2021). Also, all growth parameters were significantly lower in the positive control than in the negative control (**Figures 1d–h**), which can be attributed to the fact that no antifungal treatment was applied to the tomato plants infected by *Fusarium oxysporum*. Although the results are promising, the particle sizes reported here fall outside the typical nanoscale range (1–100 nm), which may influence their effectiveness as nanofungicides and nanofertilizers. Future studies should therefore prioritize precise size control and systematically evaluate its impact on bifunctional performance.

#### 2.1.2 Ag nanoparticles

Ag has also been studied for controlling phytopathogenic fungi and as a potential fertilizer (Ashraf et al., 2025). In this context, Ag nanoparticles synthesized using *Pongamia pinnata* leaf extract, exhibiting spherical morphology and sizes below 3 nm, were evaluated against *Fusarium oxysporum* f. sp. *lycopersici* in tomato crops under both *in vitro* and *in vivo* conditions. Additionally, their potential to enhance plant growth parameters, fruit weight and number, and bioactive compound content was assessed (Ashraf et al., 2025). The antifungal activity of Ag nanoparticles was examined under *in vitro* conditions at concentrations of 50, 100, 125, and 150 µg/mL. Fungal radial growth decreased significantly in a dose-dependent manner. Subsequently, *in vivo* experiments were conducted to evaluate their efficacy under greenhouse and field conditions. In both settings, the application of Ag nanoparticles to infected tomato plants significantly reduced disease incidence and severity. Moreover, treated plants exhibited improved growth and productivity, including increased plant height, biomass, fruit weight, and fruit number, as well as enhanced accumulation of bioactive compounds such as lycopene, flavonoids, vitamin C, and total proteins (Ashraf et al., 2025).

### 2.2 Metal oxide nanoparticles

#### 2.2.1 CuO nanoparticles

As with the metal Cu, CuO has also shown promise for controlling phytopathogenic fungi while simultaneously serving as a source of the essential micronutrient Cu. In this context, negatively and positively charged CuO nanospikes, as well as negatively charged CuO nanosheets, were synthesized via microwave-assisted hydrothermal synthesis and evaluated as nanofungicides and nanofertilizers in tomato plants via foliar application (Deng et al., 2023). Tomato plants infected with *Fusarium oxysporum* showed a significant reduction in disease progression when treated with negatively charged CuO nanosheets. Moreover, these nanosheets tended to increase shoot biomass in infected plants compared to other nanoparticle treatments. All Cu-based treatments in infected plants resulted in higher Cu accumulation in fruits relative to the control. Additionally, the concentrations of S, Ca, Mg, P, and Fe in infected tomato fruits increased due to the application of Cu nanomaterials (Deng et al., 2023). These results highlight that both nanoparticle morphology and surface charge play a fundamental role in determining their antifungal efficiency and their interaction with plant tissues, which is consistent with the well-established understanding that nanoparticle physicochemical properties govern their performance in agriculture (Cruz-Luna et al., 2021).

#### 2.2.2 ZnO nanoparticles

ZnO has also demonstrated promising potential for controlling phytopathogenic fungi while simultaneously serving as a source of the essential micronutrient Zn. In this context, commercial ZnO nanoparticles (30 ± 5 nm in particle size, spherical shape) were evaluated for controlling *Fusarium solani* and enhancing the vegetative growth and development of cherry tomato plants under greenhouse conditions at different concentrations (250, 1500, and 3000 mg/L) (Shams et al., 2023). Foliar application of ZnO nanoparticles reduced the severity of *Fusarium* wilt. Additionally, ZnO nanoparticles exhibited biostimulant effects in infected tomato, enhancing vegetative growth parameters (plant length, plant height, and number of leaves per plant), increasing leaf micronutrient concentrations (Mn, Zn, and Fe), and improving chlorophyll content and phenylalanine ammonia-lyase activity in cherry tomato leaves. The antifungal activity and biostimulant effects were dependent on nanoparticle concentration; both effects increased with nanoparticle concentration (Shams et al., 2023).

#### 2.2.3 Fe_3_O_4_ nanoparticles

The effects of positively charged (20.8 ± 2.76 nm) and negatively charged (21.00 ± 2.97 nm) Fe_3_O_4_ nanoparticles were evaluated on tomato plant growth and for controlling *Fusarium oxysporum* f. sp. *lycopersici* under both greenhouse and field conditions (Deng et al., 2024). Both positively and negatively charged Fe_3_O_4_nanoparticles significantly reduced the severity of *Fusarium* wilt by 41.4% and 44.6%, respectively, and increased shoot biomass by 327.6% and 455.0% compared with the diseased control. Moreover, positively charged Fe_3_O_4_ nanoparticles exhibited greater efficacy than their negatively charged counterparts in alleviating disease damage and modulating the accumulation of Na, Si, and Cu, highlighting the crucial role of nanoparticle surface charge in their biological performance. From an agronomic and physiological perspective, these elemental changes are relevant, as Na regulation may improve ionic homeostasis. At the same time, Si and Cu contribute to structural defense and redox-related plant immunity. Thus, their charge-dependent modulation suggests enhanced nutrient balance and stress tolerance, which is consistent with reports indicating that nanoscale iron properties, particularly particle size, control iron speciation, transport, and related metabolic responses (Li et al., 2026).

## 3 Discussion

Significant advances have been made in the bifunctional use of metal nanoparticles as both nanofungicides and nanofertilizers, with numerous studies highlighting their promising multifunctional potential across various crops (Zakharova et al., 2019; Keerthana et al., 2021; Lopez-Lima et al., 2021; Tryfon et al., 2021; Tamil Elakkiya et al., 2022; Deng et al., 2023; El-Sayed et al., 2023; Shams et al., 2023; Singh et al., 2023; Wee et al., 2023; Ajiwe and Popoola, 2024; Deng et al., 2024; Elbasuney et al., 2024; Ashraf et al., 2025; Lopez-Lima et al., 2025; Mochi et al., 2025; Saikia et al., 2026). Nevertheless, several aspects require further investigation before these bifunctional metal nanoparticles can be implemented on a large scale in agriculture.

1. Characteristics of metal nanoparticles: The bifunctional utilization of metal nanoparticles as nanofungicides and nanofertilizers is influenced by different nanoparticle characteristics, such as shape, size, crystalline structure, surface chemistry, dispersion, stability, solubility, and specific surface area (Cruz-Luna et al., 2021). In bifunctional applications, these physicochemical properties not only determine their effectiveness against phytopathogenic fungi but also regulate nutrient release, bioavailability, and interaction with plant tissues, which has been demonstrated with CuO (Deng et al., 2023) and Fe_3_O_4_ (Deng et al., 2024) nanoparticles. Nevertheless, to date, the effects of nanoparticle shapes and sizes on antifungal performance and nutrient delivery efficiency have been only partially addressed (Zakharova et al., 2019; Keerthana et al., 2021; Lopez-Lima et al., 2021; Tryfon et al., 2021; Tamil Elakkiya et al., 2022; Deng et al., 2023; El-Sayed et al., 2023; Shams et al., 2023; Singh et al., 2023; Wee et al., 2023; Ajiwe and Popoola, 2024; Deng et al., 2024; Elbasuney et al., 2024; Ashraf et al., 2025; Lopez-Lima et al., 2025; Mochi et al., 2025; Saikia et al., 2026). Therefore, it is essential to systematically investigate how size and shape influence their simultaneous role as antifungal agents and nutrient sources. Furthermore, other characteristics, including crystalline structure, surface chemistry, dispersion, solubility, stability, and specific surface area, must be carefully studied to fully understand their contributions to antifungal mechanisms, controlled nutrient release, plant uptake, and overall agronomic performance.
2. Synthesis methods: Metal nanoparticles intended for bifunctional applications as fertilizers and fungicides have been synthesized mainly through biological and chemical routes (Zakharova et al., 2019; Keerthana et al., 2021; Lopez-Lima et al., 2021; Tryfon et al., 2021; Tamil Elakkiya et al., 2022; Deng et al., 2023; El-Sayed et al., 2023; Shams et al., 2023; Singh et al., 2023; Wee et al., 2023; Ajiwe and Popoola, 2024; Deng et al., 2024; Elbasuney et al., 2024; Ashraf et al., 2025; Lopez-Lima et al., 2025; Mochi et al., 2025; Saikia et al., 2026). The biological route has gained particular attention as a greener alternative, since it uses microorganisms or plant extracts as reducing and stabilizing agents, thereby minimizing the use of hazardous chemicals and reducing environmental impact. In contrast, chemical methods may generate toxic by-products if not properly managed. However, for bifunctional applications, the key challenge is whether these synthesis strategies can produce nanoparticles with sufficiently consistent physicochemical properties to ensure reliable bifunctional performance under field conditions. In this regard, although biological synthesis offers environmental advantages, variability in biological extracts may lead to heterogeneity in size, morphology, and surface charge, potentially affecting nutrient release and antifungal efficiency. Conversely, chemical methods provide better control over these parameters, which are critical for achieving predictable nutrient delivery and pathogen inhibition, and are more suitable for large-scale production. Therefore, selecting an appropriate synthesis strategy is crucial to balance environmental sustainability with the physicochemical consistency required for effective and reproducible bifunctionality in agricultural systems.
3. Synthesis of multimetal nanoparticles: It is fundamental to produce and evaluate bimetal and trimetal nanoparticles designed for bifunctionality as both antifungal agents and nanofertilizers. Multimetal nanoparticles are particularly promising because they can exhibit improved physicochemical properties compared to their pure metal counterparts (Cruz-Martínez et al., 2021; Idris and Roy, 2023; Nyabadza et al., 2023). By combining two or more metals, it is possible to enhance not only selectivity against phytopathogenic fungi but also their fertilizing capabilities. These combinations enable synergistic effects, in which the metals interact to generate novel multifunctional properties that are not present individually (Cruz-Martínez et al., 2021; Idris and Roy, 2023; Nyabadza et al., 2023), thereby integrating disease control and crop nutrition within metal nanoparticles.
4. Environmental risks and concerns: When metal nanoparticles are proposed for bifunctional use as nanofungicides and nanofertilizers, their potential environmental and biological implications become particularly significant. Like conventional synthetic agrochemicals, the repeated and prolonged application of metal nanoparticles may pose risks to non-target organisms and negatively affect beneficial soil microorganisms, which are essential for maintaining soil fertility (Islam, 2025). Moreover, when applied at high concentrations or with unsuitable physicochemical properties, metal nanoparticles can induce phytotoxic effects, including oxidative stress, cell membrane damage, alterations in photosynthetic processes, and overall growth inhibition (Islam, 2025). Some nanoparticles may also accumulate in plant tissues and potentially enter the food chain, raising concerns about long-term ecological and human health effects (Djanaguiraman et al., 2024; Islam, 2025). In addition, leaching or runoff can transport nanoparticles into aquatic systems, where they may affect algae, invertebrates, and fish, mainly through membrane damage and oxidative stress mechanisms (Djanaguiraman et al., 2024). In this context, modern technologies are essential for addressing the environmental fate and impacts of bifunctional metal nanoparticles in agriculture. Advanced techniques, including high-resolution microscopy and spectroscopy, enable accurate detection and physicochemical characterization of nanoparticles in plant tissues, improving understanding of their uptake, translocation, accumulation, and potential entry into the food chain. Omics approaches and ecotoxicological assays further reveal phytotoxic effects and impacts on non-target organisms, including soil microorganisms and aquatic biota. Collectively, they strengthen risk assessment, guide nanoparticle design and application, and support more sustainable agriculture. Nevertheless, comprehensive studies on long-term fate, root-to-fruit translocation, ecological safety, and potential fungal resistance remain necessary before large-scale implementation.

In conclusion, metal nanoparticles exhibit strong bifunctional potential, suppressing phytopathogenic fungi while simultaneously enhancing plant growth. This bifunctionality, demonstrated across metal nanoparticles such as Cu, Ag, CuO, ZnO, and Fe_3_O_4_, highlights their promise for integrating disease control and plant nutrition in sustainable agriculture. However, key knowledge gaps remain, including the need to elucidate the role of nanoparticle physicochemical properties, assess long-term environmental and toxicity impacts, standardize field application strategies, and better understand uptake, translocation, and accumulation mechanisms in plants.

## References

[B1] Adkar-Purushothama C. R. Chettimada A. Murali T. S. Muthusamy A. Bouarab K. Perreault J. P. (2026). Non-chemical control of fungal pathogens in crops: a one-health perspective on strategies, mechanisms, and future directions. Front. Plant Sci. 16, 1746521. doi:10.3389/fpls.2025.1746521. PMID: 41602541 PMC12833398

[B2] Ajiwe S. T. Popoola A. R. (2024). Effect of copper nanoparticles on incidence and severity of Fusarium wilt and fruit yield of tomato (Solanum lycopersicum L.). Nigerian J. Bio/Technol. 41, 88–96. doi:10.4314/njb.v41i1.10

[B3] Aktar M. W. Sengupta D. Chowdhury A. (2009). Impact of pesticides use in agriculture: their benefits and hazards. Interdiscip. Toxicol. 2, 1–12. doi:10.2478/v10102-009-0001-7. PMID: 21217838 PMC2984095

[B4] Ashraf H. Anjum T. Ahmad I. S. Ahmed R. Aftab Z. E. H. Rizwana H. (2025). Phytofabricated silver nanoparticles unlock new potential in tomato plants by combating wilt infection and enhancing plant growth. Sci. Rep. 15, 10527. doi:10.1038/s41598-025-89724-4. PMID: 40148421 PMC11950516

[B5] Backer R. Rokem J. S. Ilangumaran G. Lamont J. Praslickova D. Ricci E. . (2018). Plant growth-promoting rhizobacteria: context, mechanisms of action, and roadmap to commercialization of biostimulants for sustainable agriculture. Front. Plant Sci. 9, 1473. doi:10.3389/fpls.2018.01473. PMID: 30405652 PMC6206271

[B6] Cruz-Luna A. R. Cruz-Martínez H. Vásquez-López A. Medina D. I. (2021). Metal nanoparticles as novel antifungal agents for sustainable agriculture: current advances and future directions. J. Fungi 7, 1033. doi:10.3390/jof7121033. PMID: 34947015 PMC8706727

[B7] Cruz-Luna A. R. Vásquez-López A. Rojas-Chávez H. Valdés-Madrigal M. A. Cruz-Martínez H. Medina D. I. (2023). Engineered metal oxide nanoparticles as fungicides for plant disease control. Plants 12, 2461. doi:10.3390/plants12132461. PMID: 37447021 PMC10346476

[B8] Cruz-Martínez H. Rojas-Chávez H. Matadamas-Ortiz P. T. Ortiz-Herrera J. C. López-Chávez E. Solorza-Feria O. . (2021). Current progress of Pt-based ORR electrocatalysts for PEMFCs: an integrated view combining theory and experiment. Mater. Today Phys. 19, 100406. doi:10.1016/j.mtphys.2021.100406. PMID: 38826717

[B9] Cucu M. A. Choudhary R. Trkulja V. Garg S. Matić S. (2025). Utilizing environmentally friendly techniques for the sustainable control of plant pathogens: a review. Agronomy 15, 1551. doi:10.3390/agronomy15071551. PMID: 30654563

[B10] Deng C. Protter C. R. Wang Y. Borgatta J. Zhou J. Wang P. . (2023). Nanoscale CuO charge and morphology control fusarium suppression and nutrient biofortification in field-grown tomato and watermelon. Sci. Total Environ. 905, 167799. doi:10.1016/j.scitotenv.2023.167799. PMID: 37838047

[B11] Deng C. Wang Y. Castillo C. Zhao Y. Xu W. Lian J. . (2024). Nanoscale iron (Fe_3_O_4_) surface charge controls fusarium suppression and nutrient accumulation in tomato (Solanum lycopersicum L.). ACS Sustain. Chem. Eng. 12, 13285–13296. doi: 10.1021/acssuschemeng.4c04800

[B12] Djanaguiraman M. Anbazhagan V. Dhankher O. P. Prasad P. V. (2024). Uptake, translocation, toxicity, and impact of nanoparticles on plant physiological processes. Plants 13, 3137. doi:10.3390/plants13223137. PMID: 39599346 PMC11597231

[B13] Echeverría-Pérez E. G. Cruz-López V. Herrera-Rivera R. Romellón-Cerino M. J. Rosas-Diaz J. Cruz-Martínez H. (2025). Recent developments of nanomaterials in crop growth and production: the case of the tomato (Solanum lycopersicum). Agronomy 15, 1716. doi: 10.3390/agronomy15071716

[B14] Elbasuney S. El-Sayyad G. S. Abdelaziz A. M. Rizk S. H. Tolba M. M. Attia M. S. (2024). Stable colloidal iron oxide nanoparticles: a new green nanofertilizer and therapeutic nutrient for eggplant immune response against fusarium wilt disease. J. Clust Sci. 35, 983–997. doi:10.1007/s10876-023-02527-3. PMID: 30311153

[B15] El-Sayed E. S. R. Mohamed S. S. Mousa S. A. El-Seoud M. A. A. Elmehlawy A. A. Abdou D. A. (2023). Bifunctional role of some biogenic nanoparticles in controlling wilt disease and promoting growth of common bean. AMB Express 13, 41. doi:10.1186/s13568-023-01546-7. PMID: 37119397 PMC10148937

[B16] Fasusi O. A. Cruz C. Babalola O. O. (2021). Agricultural sustainability: microbial biofertilizers in rhizosphere management. Agriculture 11, 163. doi:10.3390/agriculture11020163. PMID: 30654563

[B17] Fatima F. Hashim A. Anees S. (2021). Efficacy of nanoparticles as nanofertilizer production: a review. Environ. Sci. pollut. Res. 28, 1292–1303. doi:10.1007/s11356-020-11218-9. PMID: 33070292

[B18] Fu L. Wang Z. Dhankher O. P. Xing B. (2020). Nanotechnology as a new sustainable approach for controlling crop diseases and increasing agricultural production. J. Exp. Bot. 71, 507–519. doi:10.1093/jxb/erz314. PMID: 31270541

[B19] Gai Y. Wang H. (2024). Plant disease: a growing threat to global food security. Agronomy 14, 1615. doi:10.3390/agronomy14081615. PMID: 30654563

[B20] Hernández-Ramírez C. Rosas-Diaz J. Romellón-Cerino M. J. Priego-Clemente A. Cruz-Martínez H. (2026). Metal nanofungicides as emerging tools for crop disease control: an opinion article. Front. Soil Sci. 6, 1778571. doi: 10.3389/fsoil.2026.1778571

[B21] Idris D. S. Roy A. (2023). Synthesis of bimetallic nanoparticles and applications—an updated review. Crystals 13, 637. doi:10.3390/cryst13040637. PMID: 30654563

[B22] Islam S. (2025). Toxicity and transport of nanoparticles in agriculture: effects of size, coating, and aging. Front. Nanotechnol. 7, 1622228. doi:10.3389/fnano.2025.1622228

[B23] Keerthana P. Vijayakumar S. Vidhya E. V. N. P. Punitha V. N. Nilavukkarasi M. Praseetha P. K. (2021). Biogenesis of ZnO nanoparticles for revolutionizing agriculture: a step towards anti-infection and growth promotion in plants. Ind. Crop Prod. 170, 113762. doi:10.1016/j.indcrop.2021.113762. PMID: 38826717

[B24] Kumar Balu S. Andra S. Jeevanandam J. Kulabhusan P. K. Khamari A. Vedarathinam V. . (2023). Exploring the potential of metal oxide nanoparticles as fungicides and plant nutrient boosters. Crop Prot. 174, 106398. doi:10.1016/j.cropro.2023.106398. PMID: 38826717

[B25] Li L. J. Liu Y. F. Zhou B. Xing Z. Yuan J. Zhao T. T. (2026). Size-dependent regulation of iron transport and nitrogen metabolism by nanoscale zero-valent iron in heterotrophic nitrifying–aerobic denitrifying bacteria. Bioresour. Technol. 449, 134383. doi:10.1016/j.biortech.2026.134383 41806919

[B26] Lopez-Lima D. Carrion G. Mtz-Enriquez A. I. Duran-Barradas Z. López-Luna J. Pariona N. (2025). Two-phase Cu_x_O/Cu nanoparticles suppress Hemileia vastatrix and enhance growth in Coffea arabica plants. Bionanosci. 15, 416. doi: 10.1007/s12668-025-02036-9

[B27] Lopez-Lima D. Mtz-Enriquez A. I. Carrión G. Basurto-Cereceda S. Pariona N. (2021). The bifunctional role of copper nanoparticles in tomato: effective treatment for Fusarium wilt and plant growth promoter. Sci. Hort 277, 109810. doi:10.1016/j.scienta.2020.109810. PMID: 38826717

[B28] Mochi V. Choudhary N. Dudhagara D. Yadav V. K. Patel A. (2025). Green synthesis of Cu‐Fe nano bimetallic particles: a dual approach for crop protection and growth stimulation in Vigna radiata. Appl. Organomet. Chem. 39, e70223. doi:10.1002/aoc.70223. PMID: 41531421

[B29] Ndaba B. Roopnarain A. Rama H. Maaza M. (2022). Biosynthesized metallic nanoparticles as fertilizers: an emerging precision agriculture strategy. J. Integr. Agric. 21, 1225–1242. doi:10.1016/s2095-3119(21)63751-6

[B30] Nyabadza A. McCarthy É. Makhesana M. Heidarinassab S. Plouze A. Vazquez M. . (2023). A review of physical, chemical and biological synthesis methods of bimetallic nanoparticles and applications in sensing, water treatment, biomedicine, catalysis and hydrogen storage. Adv. Colloid Interface Sci. 321, 103010. doi:10.1016/j.cis.2023.103010. PMID: 37804661

[B31] Ogwu M. C. Izah S. C. (2025). Nanotechnology for fungal pathogen control in crops: innovations, public health impacts, and disease prevention. Front. Fungal Biol. 6, 1653214. doi:10.3389/ffunb.2025.1653214. PMID: 40959466 PMC12434137

[B32] Paramesh V. Mohan Kumar R. Rajanna G. A. Gowda S. Nath A. J. Madival Y. . (2023). Integrated nutrient management for improving crop yields, soil properties, and reducing greenhouse gas emissions. Front. Sustain. Food. Syst. 7, 1173258. doi:10.3389/fsufs.2023.1173258

[B33] Rani L. Thapa K. Kanojia N. Sharma N. Singh S. Grewal A. S. . (2021). An extensive review on the consequences of chemical pesticides on human health and environment. J. Clean. Prod. 283, 124657. doi:10.1016/j.jclepro.2020.124657. PMID: 38826717

[B34] Raza A. Khandelwal K. Pandit S. Singh M. Kumar S. Rustagi S. . (2024). Exploring the potential of metallic and metal oxide nanoparticles for reinforced disease management in agricultural systems: a comprehensive review. Environ. Nanotechnol. Monit. Manag 22, 100998. doi:10.1016/j.enmm.2024.100998. PMID: 38826717

[B35] Saikia D. Baruah P. K. Sarmah S. R. Prasad R. Sarma H. (2026). Zinc oxide nanoparticles enable sustainable disease management in tea by dual nutrient and antifungal action. Open Life Sci. 21, 20251260. doi:10.1515/biol-2025-1260. PMID: 41717542 PMC12915709

[B36] Sanguiñedo P. Faccio R. Abreo E. Alborés S. (2023). Biogenic silver and copper nanoparticles: potential antifungal agents in rice and wheat crops. Chemistry 5, 2104–2119. doi: 10.3390/chemistry5040143

[B37] Shams A. H. Helaly A. A. Algeblawi A. M. Awad-Allah E. F. (2023). Efficacy of seed-biopriming with trichoderma spp. and foliar spraying of ZnO-nanoparticles induce cherry tomato growth and resistance to Fusarium wilt disease. Plants 12, 3117. doi:10.3390/plants12173117. PMID: 37687362 PMC10489679

[B38] Shashidhara V. Bhattacharjee S. Alwarsamy M. (2025). Boosting agronomic yields with metallic nano-fertilizers: an effective approach for sustainable agricultural development. Commun. Soil Sci. Plant Anal. 56, 1734–1750. doi:10.1080/00103624.2025.2468250. PMID: 37339054

[B39] Singh D. Jain D. Rajpurohit D. Jat G. Kushwaha H. S. Singh A. . (2023). Bacteria assisted green synthesis of copper oxide nanoparticles and their potential applications as antimicrobial agents and plant growth stimulants. Front. Chem. 11, 1154128. doi:10.3389/fchem.2023.1154128. PMID: 37090246 PMC10119401

[B40] Tamil Elakkiya V. Meenakshi R. V. Senthil Kumar P. Karthik V. Ravi Shankar K. Sureshkumar P. . (2022). Green synthesis of copper nanoparticles using Sesbania aculeata to enhance the plant growth and antimicrobial activities. Int. J. Environ. Sci. Technol. 19, 1313–1322. doi:10.1007/s13762-021-03182-9. PMID: 30311153

[B41] Tryfon P. Kamou N. N. Mourdikoudis S. Karamanoli K. Menkissoglu-Spiroudi U. Dendrinou-Samara C. (2021). CuZn and ZnO nanoflowers as nano-fungicides against Botrytis cinerea and Sclerotinia sclerotiorum: phytoprotection, translocation, and impact after foliar application. Materials 14, 7600. doi:10.3390/ma14247600. PMID: 34947215 PMC8708589

[B42] Wee J. L. Chan Y. S. Law M. C. (2023). Dual functions of a hybrid magnetic magnesium oxide nanocomposite as a fungicide and plant growth promoter in agriculture applications. ACS Appl. Bio Mater. 6, 4972–4987. doi:10.1021/acsabm.3c00515. PMID: 37910790

[B43] Zakharova O. Kolesnikov E. Shatrova N. Gusev A. (2019). The effects of CuO nanoparticles on wheat seeds and seedlings and Alternaria solani fungi: *in vitro* study. IOP Conf. Ser. Earth Environ. Sci. 226, 12036. doi:10.1088/1755-1315/226/1/012036

